# Formulation and Performance of Model Concrete in Reduced-Scale Physical Model Tests

**DOI:** 10.3390/ma16175784

**Published:** 2023-08-24

**Authors:** Gang Zheng, Boyang Xia, Haizuo Zhou, Yu Diao, Jianyou Huang, Junbo Zhang, Xiaoxuan Yu

**Affiliations:** 1 School of Civil Engineering, Tianjin University, Tianjin 300072, China; zhenggang1967@163.com (G.Z.); boyang_1027@tju.edu.cn (B.X.); yudiao@tju.edu.cn (Y.D.); jianyou_huang@163.com (J.H.); zhangjunbo@tju.edu.cn (J.Z.); yuxiaoxuan@tju.edu.cn (X.Y.); 2 Key Laboratory of Coast Civil Structure Safety, Tianjin University, Ministry of Education, Tianjin 300072, China; 3 State Key Laboratory of Hydraulic Engineering Simulation and Safety, Tianjin University, Tianjin 300072, China

**Keywords:** model concrete, small-scale physical test, α-gypsum plaster, hydration, mechanical strength, prediction model

## Abstract

The utility of geotechnical centrifuge tests depends on how correctly they predict the physical and mechanical behaviour of concrete. In this study, a model concrete material that consisted of α-gypsum plaster, fine silica sand, and water was developed. An orthogonal test design was used to evaluate the effect of the mix proportion on the model concrete performance. The physical (i.e., flowability and bleeding rate) and mechanical (i.e., compressive and flexural strength) characteristics were considered as indices. Various mix ratios resulted in remarkable relative contributions to model concrete performance, and each raw material dosage exhibited positive or negative synergy. The water–plaster ratio (W/P) and aggregate–plaster ratio (A/P) strongly influenced the mechanical and physical characteristics, respectively. Multiple linear regression analysis (MLRA) was carried out to determine a forecast model for various small-scale test demands. Finally, the applicability and outlines of the presented forecasting method in proportioning design were evaluated by typical use of model concrete in small-scale model tests.

## 1. Introduction

Simple concrete, which has relatively high strength and stiffness, is an efficient and effective solution for geotechnical infrastructure in soft soil [1,2,3,4,5]. In addition to full-scale field testing, small-scale modelling is pertinent to investigating the mechanical and failure behaviour of simple concrete structures and soil–structure interactions [6,7,8,9]. Appropriate modelling of the prototype stress level is crucial in small-scale physical tests for capturing the stress-dependent characteristics in geotechnical engineering. Centrifuge modelling can produce a prototype stress by using centrifugal acceleration (g). On this basis, researchers can test small-scale physical models at stress levels identical to those experienced at field-prototype scale [10]. To reliably simulate the behaviour of concrete structures in small-scale model tests, the material properties of the element should be correctly scaled. However, the particle size of coarse aggregates in concrete is difficult to scale accurately (e.g., for the 1:40 scale, a 5 mm grain diameter of the aggregate is the same as the size of a 0.2 m prototype), which overestimates the strength of the model material. Furthermore, conventional simple concrete usually requires 28 d to reach the expected strength, which is inefficient for model tests.

A gypsum-based mixture mortar is useful in small-scale modelling and can reflect the quasi-brittle nature of concrete; especially in centrifuge model tests, which require large-scale factors [11,12]. Knappett et al. [11] evaluated various types of gypsum-based mixture mortar and uniformly graded silica sand, which they used to geometrically scale the coarse aggregate. Such model concrete was able to simulate the quasi-brittle failure behaviour of concrete and exhibited typical mechanical strengths. This type of mixture mortar was recently used in modelling concrete in centrifuge model tests [13,14,15]. However, the formulations of model concrete in literature studies are only applicable to specific test conditions and cannot be extended in generalized applications. Generally, excellent performance of mixture mortar requires favourable fluidity, stability, and sufficient mechanical strength [16,17,18]. Therefore, an approach that can describe the relationship between the various mix proportions and performance is necessary; a quantitative analysis should be applied to evaluate the effects of each mix factor and determine the optimal formulations for use in different small-scale tests.

In this study, a total of 20 groups of gypsum-based mortar samples with various water–plaster ratios and silica sand dosages were tested by orthogonal test design. The flowability, bleeding rate, and compressive and flexural strength were considered as evaluation indices of model concrete materials. Prediction models of the relationship between the evaluation indices and various mix proportions are proposed by using multiple linear regression analysis (MLRA). Finally, by typical application in a centrifuge model test, guidance for general application of gypsum-based materials for modelling concrete elements in small-scale physical tests are provided.

## 2. Raw Materials and Methodologies

### 2.1. Raw Materials

**Figure 1:**
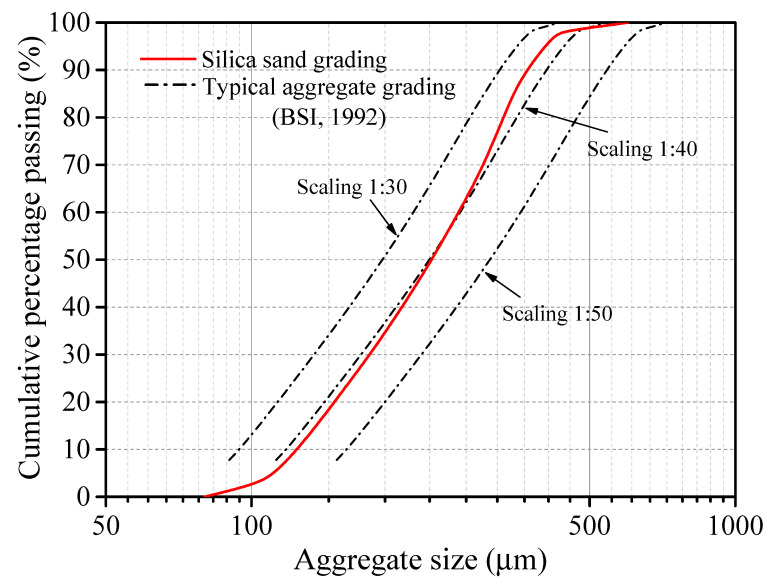
Particle size distributions of coarse aggregate (fine silica sand) [20].

To satisfy the spatial and physical scaling laws between small-scale test and those of a corresponding prototype, the raw materials of the emulation model concrete column consisted of α-gypsum plaster (model concrete; Jing-men, Hubei, China), water, and fine silica sand (coarse aggregate; Zhengzhou, Henan, China). The chemical composition of the α-gypsum plaster was analysed with an X-ray fluorescence spectrometer (model Panalytical Axios, Malvern Panalytical, Malvern, UK) [19], as shown in Table 1 and Table 2. Figure 1 shows the grading curve of the fine silica sand. The particle size distribution was consistent with a typical coarse aggregate grading specified in BS 882 [20], scaled between 1:30 and 1:50.

### 2.2. Orthogonal Test Approach

To investigate the effect of various mixing ratios on the performance of model concrete, an orthogonal test design was used. The advantage of orthogonal testing was to select representative test points from a comprehensive test plan for minimizing the experimental quantity and obtaining comprehensive test results. Thus, the water/plaster (W/P) ratio and coarse aggregate content were considered as the pertinent variables in the orthogonal tests. Remarkably, the mass fraction of plaster (A/P) was used as the content of fine silica sand, which served as the coarse aggregate for model concrete. In accordance with relevant research and previous experiments, the standards of each mixing ratio were determined by choosing typical values [11]. Table 3 shows the experiment composition of 20 groups of test samples. The influence of the W/P and A/P ratios on the flowability, bleeding, and compressive and flexural strength performance is discussed in subsequent paragraphs.

### 2.3. Preparation of Model Concrete Specimens

All 20 groups of model concrete mortar samples (Table 3) consisted of α-gypsum plaster, fine silica sand, and water. The preparation process was as follows.

The weighted powder, including the alpha-gypsum plaster and fine silica sand, was added into the mixer and dry-mixed for about 60 s.

The sand plaster mixture was poured into the water and quickly stirred in the mixer for 120 s.

Fresh paste and casted samples were prepared for further testing.

### 2.4. Test Methods

**Figure 2:**
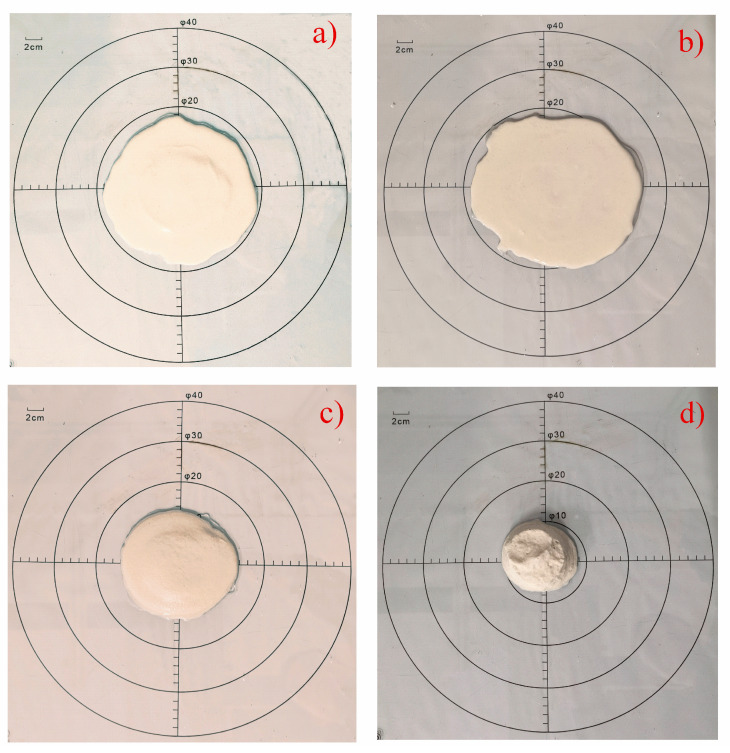
Flowability characteristic of mini-slump tests: ((**a**) A2; (**b**) B2; (**c**) C5; (**d**) A5).

The flowability characteristic of the model concrete mortar was determined by measuring the spread diameter of fresh mixture slurry by a mini-slump test [21,22]. A 40 × 40 cm glossy glass plate was used in the test and a typical steel mould was placed in the centre of the glossy glass. The mould filled with the slurry was rapidly lifted from the glass plate and then the slurry flowed freely onto the glass plate for measuring the maximum expanded diameter length of the slurry. Figure 2 shows typical results of mini-slump tests.

**Figure 3:**
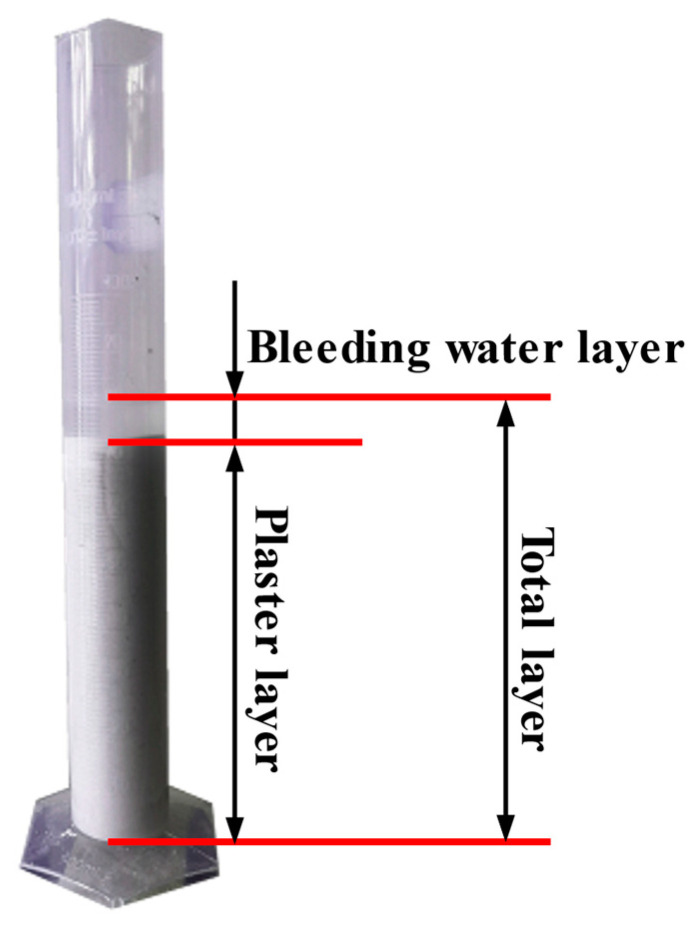
Schematic of bleeding rate test.

The standing observation method indicates the stability of the mixture paste by measurement of the percentage of the upper bleeding water volume of fresh slurry compared with the total volume (Figure 3). A larger water bleeding ratio corresponds to lesser stability of the fresh mixture slurry [17,18,23].

**Figure 4:**
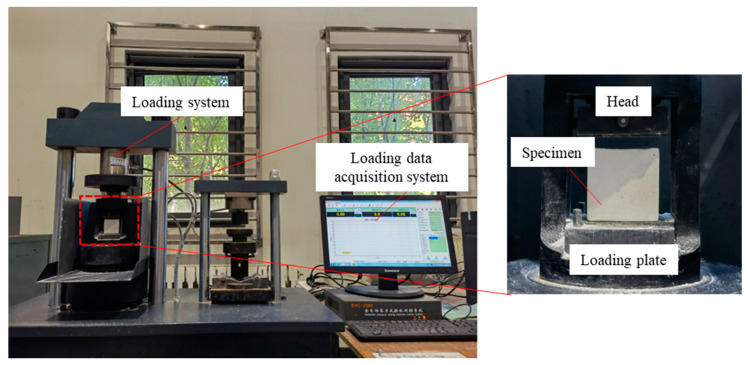
Schematic of unconfined compressive strength test system.

Cubic model concrete specimens of a standard size (50 × 50 × 50 mm) were prepared for unconfined compression tests. The specimens were made by pouring the sand plaster mixture into the mould and were subsequently demoulded. Afterwards, the standard cubes were cured for 3, 7, 14, and 28 days under standard curing conditions at a temperature of 25 ± 2 °C and a relative humidity of 95% [24]. The compressive strength of the specimens was tested with an MTS servo-hydraulic testing machine under a loading of 2.4 kN/s (Figure 4) [23,24].

**Figure 5:**
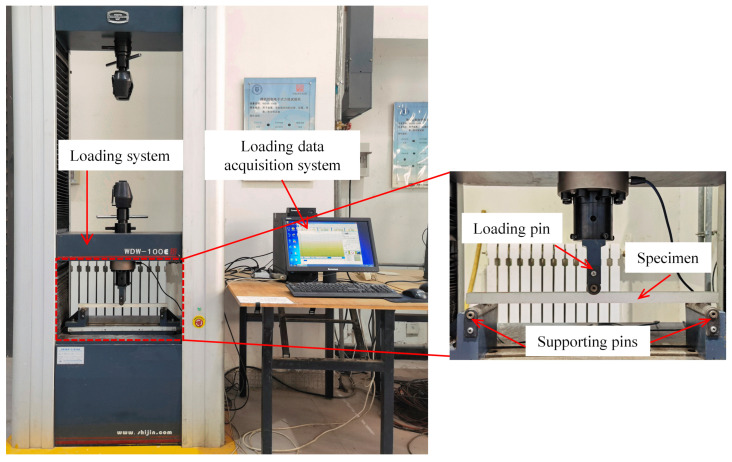
Schematic of three-point bending test.

Compared with the direct flexural strength using standard specimens, the modulus of rupture (*f*_r_) was considered to be more suitable for representing the flexural behaviour of concrete under bending-induced flexural stress [11,25,26]. The *f*_r_ has a substantial size effect. For each bending test, prismoid-shaped specimens (size: 20 mm × 20 mm × 450 mm) were used to obtain the average *f*_r_ for each mixing composition. These dimensions can provide corresponding values of *f*_r_ for subsequent centrifuge model tests of a model concrete pile at 1:40 scale [26]. Figure 5 shows installation details of the test. Bending tests were performed with a microcomputer-controlled electronic universal testing machine (model WDW-100E); vertical concentrated load was applied in a displacement-controlled mode (0.5 mm/min) until specimen rupture [27].

## 3. Orthogonal Experiment Results and Correlation Analysis

### 3.1. Flowability

**Figure 6:**
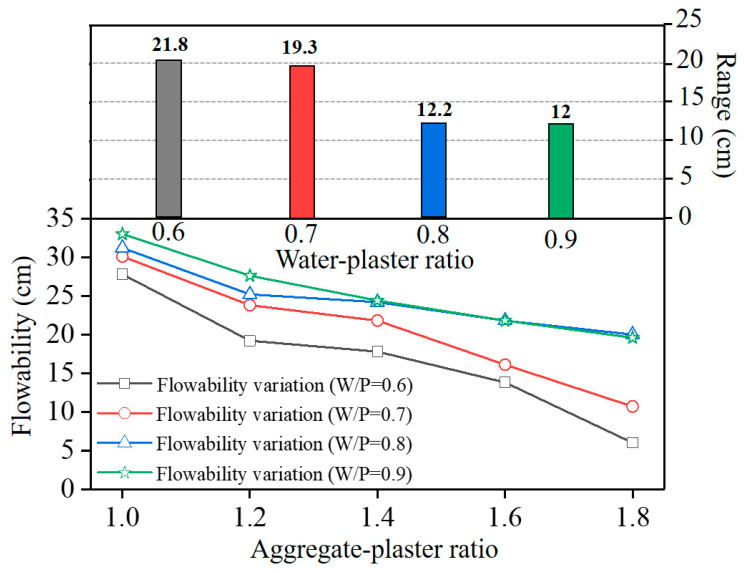
Influence of each composition on the flowability of the mixture slurry.

Flowability is a significant index for fresh mixture mortar and must be sufficiently large to meet the requirements of homogeneity [28,29]. Figure 6 shows the influence of the A/P ratio on the flowability of mixture slurries with various W/P ratios. The flowability decreased gradually with increasing A/P ratio, and more substantially so at lower W/P ratios (e.g., W/P = 0.6, 0.7). At a given A/P ratio, the flowability depended on the W/P ratio; the aggregate content required to achieve a decreasing flowability was lower with decreasing W/P ratio. In accordance with the range analysis, the significance of the influence of the aggregate content on the flowability decreased with the increasing W/P ratio. Higher W/P ratios led to increasing relative content of free water in the slurry, and the free water caused an increase in the overall flowability by reducing the friction contact among the particles [18]. As a result, the mixing proportion of the maximum flowability of the slurry was W/P = 0.9 and A/P = 1.

### 3.2. Bleeding

**Figure 7:**
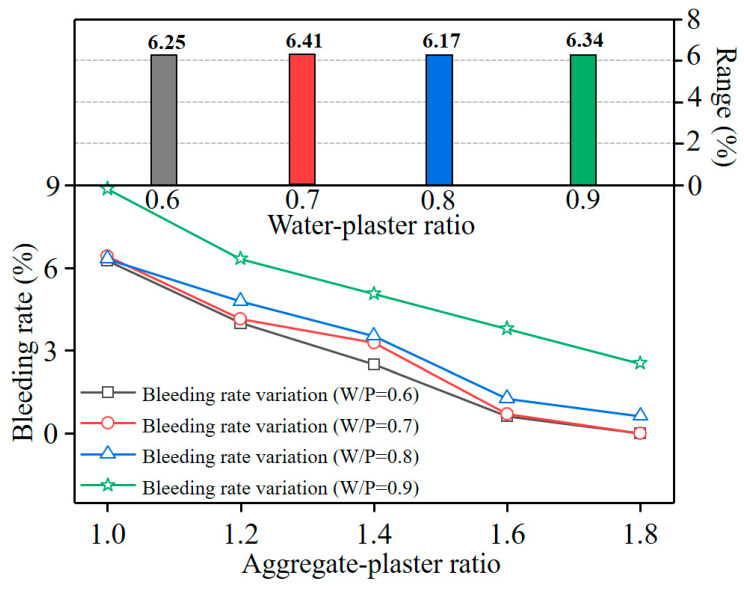
Influence of each composition on the bleeding rate of the mixture slurry.

Bleeding refers to separation of water from the pores between filled particles. A higher bleeding rate results in instability of the mixture mortar, which leads to large volume change of the specimen during hardening and deviation from the design volume [25]. Figure 7 shows the variation of the bleeding rate for all the specimens with various ratios. The bleeding rate decreased with increasing A/P ratio. This result is possibly because free water in the paste was absorbed by sand particles with a large specific surface area. Therefore, the stability of the slurry can be improved by reducing the content of water in the formulation. When the A/P ratio exceeded 1.6, the effect of the aggregate content on reducing the bleeding rate lessened. Moreover, the range analysis indicates that the significance of the impact of the A/P ratio on the bleeding rate was almost invariable under various W/P ratios.

### 3.3. Compressive Strength

**Figure 8:**
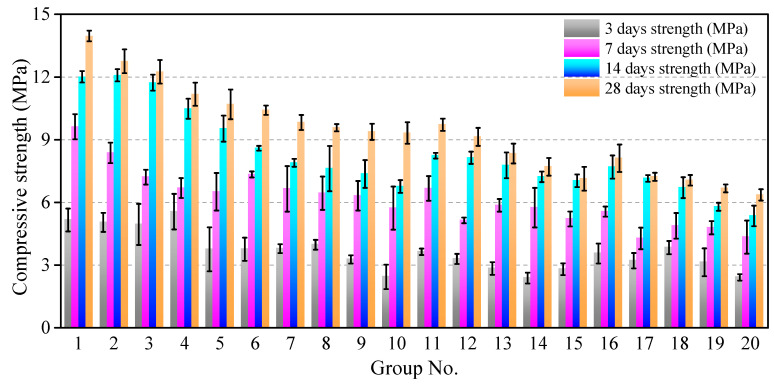
Variation in compressive strength of specimens from curing 3–28 days.

Figure 8 shows the variation in the compressive strength of all of the specimens from curing 3–28 days. A remarkable compression strength diversity was found between 3 and 7 days for all sets of test specimens. For example, regarding group No. 6, the 3 d compressive strength value only accounted for 51.2% of the 7 d compressive strength. In addition, the compressive strength exhibited a significant correspondence to the W/P ratio; the specimens with W/P = 0.6 (no. 1–5) exhibited the highest compressive strength after 28 days of curing.

**Figure 9:**
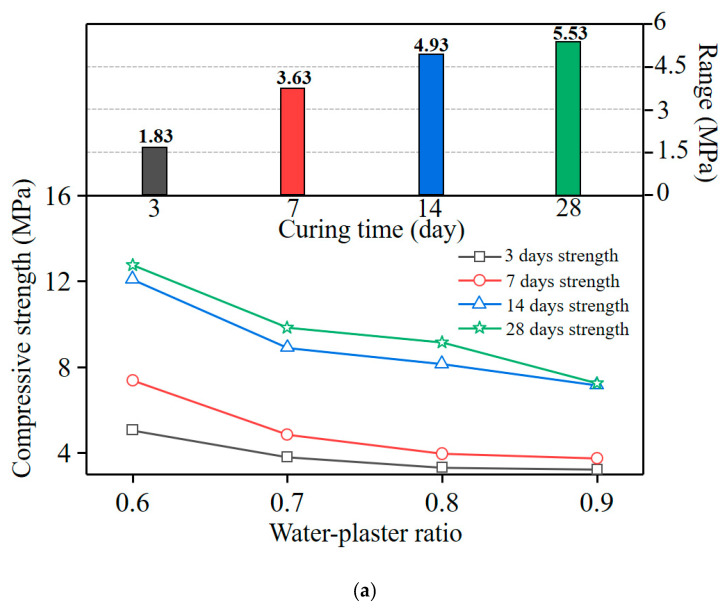

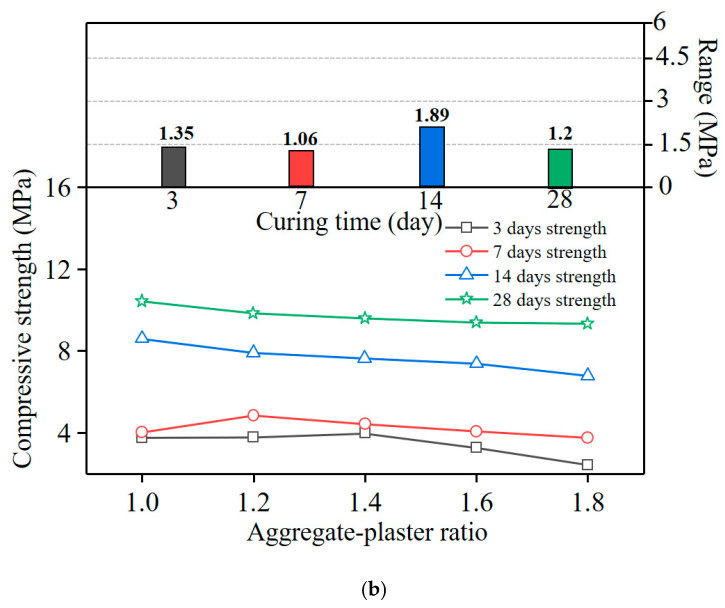
Influence of each mix proportion on the compressive strength: (**a**) water/plaster ratio; (**b**) aggregate/plaster ratio.

Figure 9 shows the influence of pertinent variables on the compressive strength. The compressive strength increased significantly with the decreasing W/P ratio, whereas the A/P ratio exhibited a negligible effect on the compressive strength. This phenomenon is because the corresponding content of plaster particles in the paste increased with smaller water/plaster ratios, and the active ingredients in the plaster formed more high-strength hydration products after adequate hydration, which led to an increase in the compressive strength [19,30]. The optimum for the 28 d compressive strength of the sample was group No. 1. The range analysis results indicate that the significance of each composition on compressive strength was (R_W/P_) > (R_A/P_).

### 3.4. Flexural Strength

**Figure 10:**
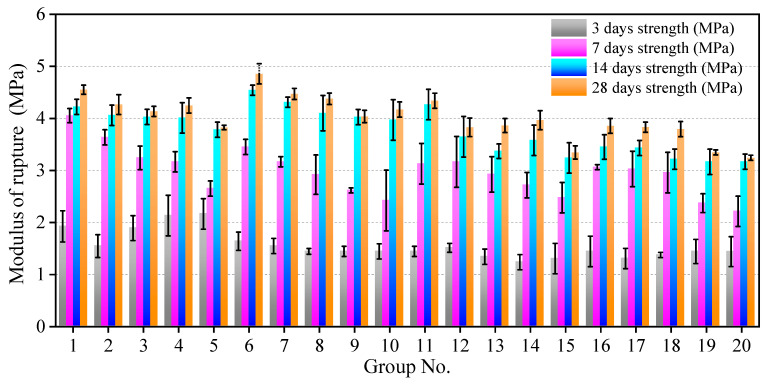
Variation in flexural strength of specimens from curing 3–28 days.

To describe the behaviour of the concrete under bending-induced flexural stress (i.e., flexural strength of the model concrete), the modulus of rupture was used [31]. Figure 10 shows the variation of the modulus of rupture from curing 3–28 days. Compared with the compressive strength, the flexural strength of the model concrete was relatively low, which is in accordance with a quasi-brittle material that exhibits low flexural fracture energy. After curing for 14 days, the modulus of rupture value tended to be stable, indicating that the hardening of the model concrete was nearly complete.

**Figure 11:**
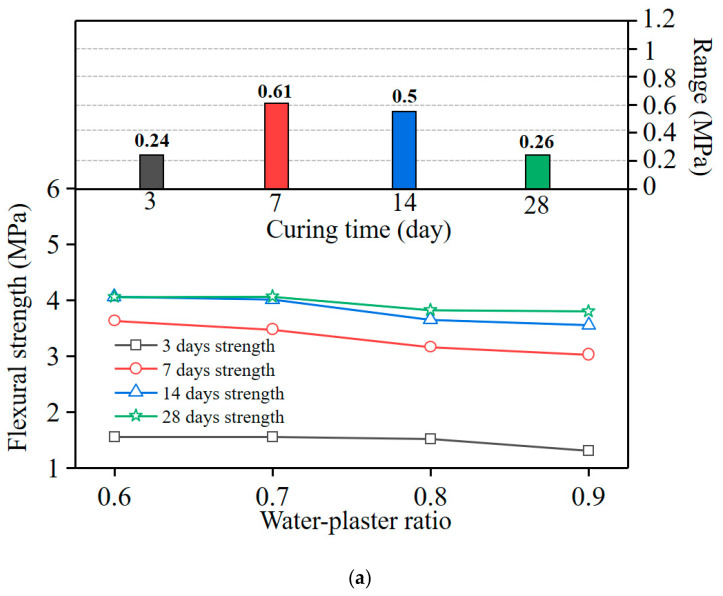

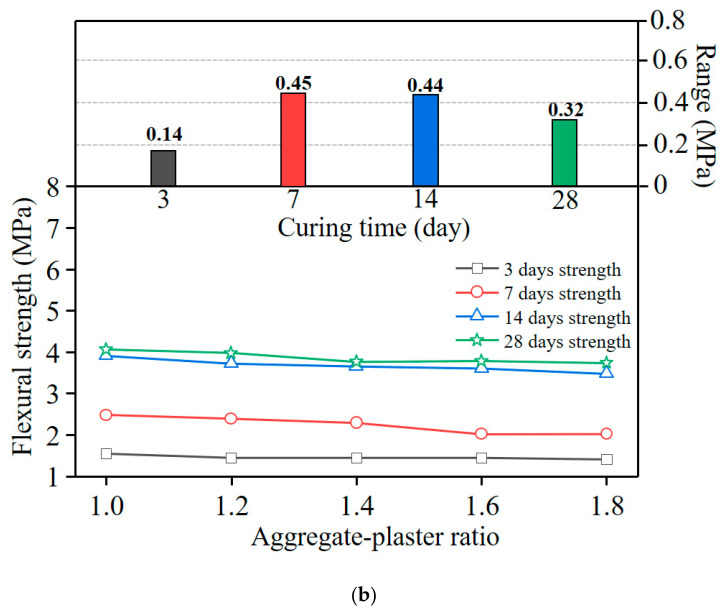
Influence of each variable on the flexural strength: (**a**) water/plaster ratio; (**b**) aggregate/plaster ratio.

Figure 11 shows the effects of pertinent variables on the flexural strength. After curing for 3 days, the influence of the mix proportion on the flexural strength was slight; the maximum range differences of the W/P ratio and A/P ratio were 0.24 and 0.14 MPa, respectively. With increasing curing time, the influence of each composition on the flexural strength became obvious. After curing for 28 days, the significance of each composition on the flexural strength lessened, illustrating that the hydration was nearly complete in the specimens and stable strength was achieved.

### 3.5. Correlation Analysis

The correlation between various indices in this study was further investigated by Pearson correlation analysis [32]. Pearson’s correlation coefficient is defined as

(1) $$\rho \left(X,Y\right)=\frac{\mathrm{cov}\left(X,Y\right)}{\sigma_{X}\sigma_{Y}}$$

where ρ is the population correlation coefficient; *X*, *Y* are the two datasets of indices; and σ_x_ and σ_y_ are the standard deviation of *X*, *Y*, respectively. A large coefficient indicates a significant relationship between the two indices.

**Figure 12:**
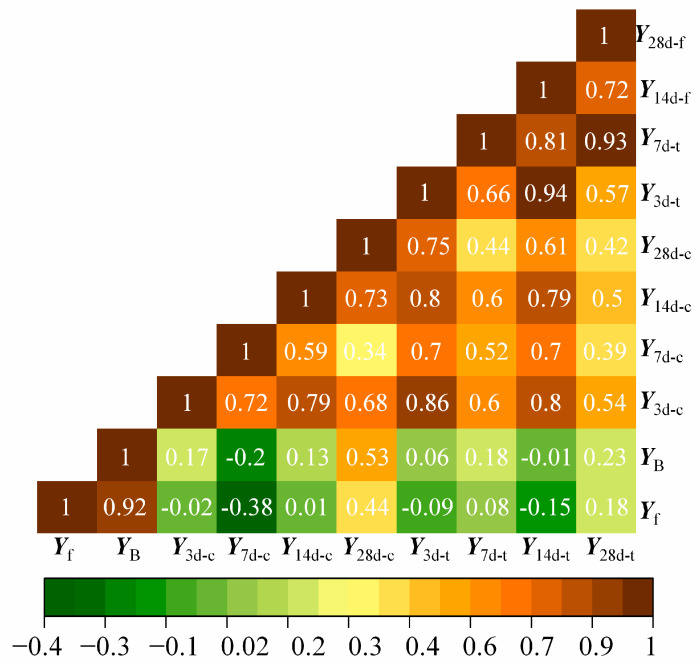
Heatmap of Pearson’s correlation coefficients.

Figure 12 shows a heatmap of the interrelation between each index. A Pearson’s coefficient value of |ρ| > 0.7 indicates a significant correlation, 0.4 < |ρ| < 0.7 indicates a medium correlation, and |ρ| < 0.4, indicates a weak correlation [33]. The aforementioned correlation analysis demonstrates that the correlation between the flowability and bleeding rate was significant (Pearson’s coefficient: 0.92). This phenomenon is attributable to the fact that the flowability and bleeding rate are determined by the free water content in the mixture paste. For the mechanical properties, there is a positive correlation between the compressive and flexural strength at different curing times. The highest correlation between these two strength indicators is observed after a curing period of 14 days, with a corresponding Pearson correlation coefficient of 0.79. Furthermore, there is a relatively weak correlation between the physical and mechanical characteristics of the model concrete, indicating that there is no inherent connection between them and they are independent of each other.

## 4. MLRA

The significance of each composition on the performance indices could be determined by an analysis of the orthogonal test results, and then the most appropriate ratio can be selected to simulate the concrete structure behaviour in small-scale models. However, the specific mixing ratio of model concrete is frequently modified to satisfy the requirements for various small-scale model test conditions. To conveniently determine the optimum mixing ratio, MLRA was performed to forecast the ratio parameters of the model concrete in this study. SPSS statistics (version 26; IBM, Armonk, NY, USA) were used to develop the predicting regression models by MLRA by 20 set results of orthogonal tests.

### 4.1. Validation of MLRA Models

To verify the feasibility of the MLRA models developed in this study, all of the test indices (flowability; bleeding rate; 3-, 7-, 14-, and 28 days compressive strength; and 3-, 7-, 14-, and 28 days flexural strength) should be statistically verified. Table 4 shows the correlation coefficients between the dependent variables (orthogonal test results) and the independent variable (W/P and A/P ratios). The fitted linear regression coefficients (R^2^) reveal excellent linear relationships between the independent and dependent variables. Moreover, the significance of variance analysis in Table 5 was all <0.05, confirming the highly statistical significance.

### 4.2. Multiple Regression Coefficients

Table 5 shows the results of the regression coefficients; the importance of various mix proportions on the indices can be revealed by standardized regression coefficients. For example, in the model *F*_flowability_, the standardized coefficients of the A/P and W/P ratios were −0.82 and 0.48, respectively. Thus, the aggregate contents negatively influenced the flowability of the slurry, whereas the water/plaster ratio exhibited positive synergy. In addition, the significance of the two mix proportions on the flowability—which considers the absolute value of standardized coefficients—was A/P > W/P, and it is consistent with the orthogonal test result analysis. On the basis of the regression coefficients in Table 5, linear relationship models between the mix proportion and indices of model concrete were developed. For example, the relationships between flowability, bleeding rate, and 7 days compressive and flexural strength with various compositions of model concrete were proposed as

(2) $$F_{\mathrm{flowability}}(\mathrm{cm})=27.3+26.67\times W/P-18.09\times A/P$$

(3) $$F_{\mathrm{bleeding}}(\%)=8.2+8.33\times W/P-7.78\times A/P$$

(4) $$F_{7-d\;\mathrm{compressive}}\;(\mathrm{MPa})=18.038-9.499\times W/P-1.962\times A/P$$

(5) $$F_{7-d\;\mathrm{flexural}}\;(\mathrm{MPa})=6.22-1.942\times W/P-1.268\times A/P$$

where *F*_flowability_, *F*_bleeding_, *F*_7-d compressive_, and *F*_7-d flexural_ are the prediction equations of the flowability, bleeding rate, and 7 days compressive and flexural strength of model concrete, respectively.

## 5. Guidelines for the Optimal Parameter Selection

To verify the feasibility of the prediction model and further explain the guidelines for parameter selection of the model concrete for small-scale modelling, a typical application of model concrete piles in a geotechnical centrifuge test is presented. The aim of the presented centrifuge test is to investigate the failure behaviour of a concrete column-supported embankment. In general, concrete columns undergo bending failure in practice, which causes global failure of the embankment [5,34]. Therefore, the flexural strength of model concrete is the most important performance index and must satisfy the requirements put forward by the design criterion in prototypes. The target flexural strength of the model concrete in this centrifuge test case was about 2.81 MPa and the bleeding rate was ≤ 5%, which satisfies engineering criteria [5,34,35,36,37]. Combined with predicted model and orthogonal test results, two formulations (W/P = 0.7; A/P = 1.6 and W/P = 0.8; A/P = 1.4) that satisfied the requirements were selected.

**Figure 13:**
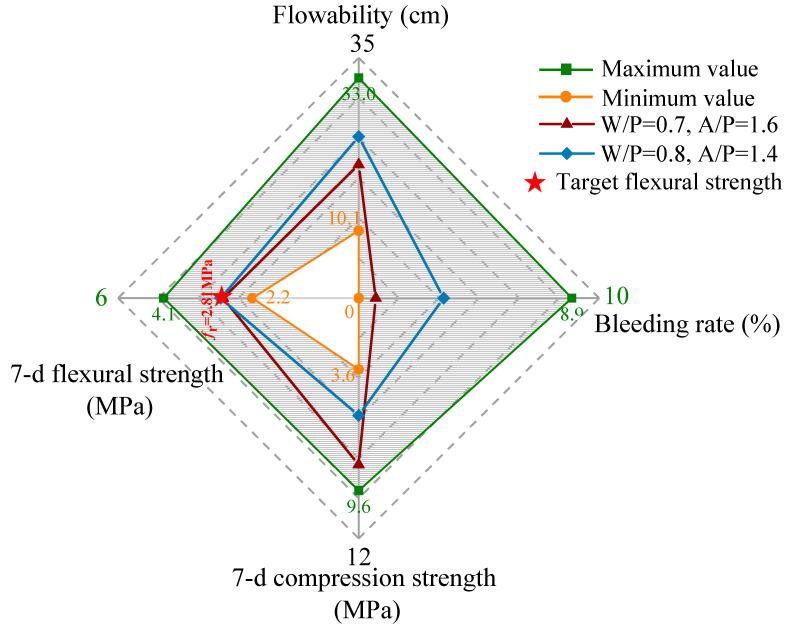
Range of optional aggregate dosages and W/P ratios for target flexural strength.

Figure 13 shows the relationship between the model concrete performance and the minimum–maximum index values of the orthogonal tests. Considering the multi-performance criteria of model concrete, such as high flowability, good structural stability (low bleeding rate), and sufficient compressive strength, the recommended water/plaster and aggregate/plaster ratios by the prediction model are 0.7 and 1.6, respectively. The optimal formulation achieves sufficient flexural strength after 7 days of curing, whereas other indices could also meet the material performance requirements.

## 6. Conclusions

In this study, orthogonal test design was applied to quantitatively evaluate the effects of various compositions on model concrete performance. On the basis of the test results, a forecast model for determining the optimal formulations of model concrete was proposed to satisfy various small-scale model test requirements. The following conclusions were drawn.

(i) The effects of aggregate content and water/plaster ratio on the performance of model concrete were interdependent. The aggregate content negatively influenced the flowability and the bleeding rate of the slurry, whereas the water/plaster ratio exhibited positive synergy. Increasing the aggregate content and water/plaster ratio negatively corresponded to the mechanical characteristics, which was pertinent to compressive and flexural strength.

(ii) The correlation between the flowability and bleeding rate was significant (Pearson correlation coefficient: 0.92) because of the high content of free water in the mixture slurry. Moreover, the correlation between the compressive and flexural strength was positive; a significant correlation coefficient was found after curing for 14 days.

(iii) On the basis of the MLRA, a prediction model for the performance of model concrete was proposed. The significance level of each mix was proposed in the following sequence: for optimum flowability and bleeding rate, A/P > W/P. However, regarding the compressive and flexural strength, A/P < W/P.

(iv) Outlines for parameter selection of the model concrete were summarized based on a typical application case in geotechnical centrifuge testing. Combined with the specific test target, a reasonable range of water/gypsum ratios and aggregate content can be precisely determined by the prediction model.

## Figures and Tables

**Table 1:** Chemical compositions of α-gypsum plaster (wt%).

Material	MgO	Al_2_O_3_	SiO_2_	SO_3_	CaO	SrO
α-gypsum	1.28	0.084	0.143	38.291	59.605	0.597

**Table 2:** Physical properties of α-gypsum plaster.

Material	Consistency (%)	Initial Setting Time (min)	Final Setting Time (min)	Bulk Density (kg/m^3^)	7 Days Compressive Strength (MPa)
α-gypsum	58.0	8	17	1200	15

**Table 3:** Composition of the orthogonal tests.

Parameters		Water Quantity (g)	Plaster Quantity (g)	Aggregate Quantity (g)	W/P	A/P
	A1	300	500	500	0.6	1
	A2	300		600		1.2
A	A3	300		700		1.4
	A4	300		800		1.6
	A5	300		900		1.8
	B1	350	500	500	0.7	1
	B2	350		600		1.2
B	B3	350		700		1.4
	B4	350		800		1.6
	B5	350		900		1.8
	C1	400	500	500	0.8	1
	C2	400		600		1.2
C	C3	400		700		1.4
	C4	400		800		1.6
	C5	400		900		1.8
	D1	450	500	500	0.9	1
	D2	450		600		1.2
D	D3	450		700		1.4
	D4	450		800		1.6
	D5	450		900		1.8

**Table 4:** Description of fitting models.

Model	R	R^2^	Adjusted R^2^	Std. Error of the Estimate
F_flowability_	0.956	0.914	0.904	1.983
F_bleeding_	0.933	0.871	0.855	0.992
F_3-d compressive_	0.972	0.945	0.939	0.509
F_3-d flexural_	0.85	0.722	0.69	0.2539
F_7-d compressive_	0.926	0.858	0.841	0.916
F_7-d flexural_	0.958	0.91	0.907	0.136
F_14-dcompressive_	0.877	0.769	0.723	1.172
F_14-d flexural_	0.846	0.715	0.679	0.367
F_28-d compressive_	0.884	0.781	0.724	0.61
F_28-d flexural_	0.843	0.71	0.677	0.188

**Table 5:** Results of regression coefficients.

Model		Unstandardized Coefficients		Standardize Coefficients (Beta)	Statistic (t)	Sig.
		Regression Coefficients	Std. Error			
F_flowability_	Constant	27.30	3.805	/	7.715	<0.001
	A/P	−18.09	1.602	−0.82	−11.29	<0.001
	W/P	26.67	4.054	0.48	6.58	<0.001
F_bleeding_	Constant	8.20	1.219	/	6.73	<0.001
	A/P	−7.78	0.513	−0.89	−15.16	<0.001
	W/P	8.33	1.298	0.38	6.41	<0.001
F_3-d compressive_	Constant	9.54	1.147	/	8.32	<0.001
	A/P	−1.30	0.483	−0.41	−2.69	0.015
	W/P	−5.44	1.222	−0.67	−4.45	<0.001
F_3-d flexural_	Constant	2.82	0.355	/	6.07	<0.001
	A/P	0.02	0.387	−0.33	−1.85	0.031
	W/P	−1.73	0.149	−0.61	−3.46	0.003
F_7-d compressive_	Constant	18.038	1.72	/	12.89	<0.001
	A/P	−1.962	0.724	−0.33	−3.47	0.003
	W/P	−9.499	1.832	−0.61	−7.56	<0.001
F_7-d flexural_	Constant	6.22	0.256	/	8.09	<0.001
	A/P	−1.942	0.108	−0.819	−7.11	<0.001
	W/P	−1.268	0.273	−0.496	−11.74	<0.001
F_14-d compressive_	Constant	22.16	0.355	/	7.96	<0.001
	A/P	−2.51	0.149	−0.37	0.12	0.003
	W/P	−13.85	0.378	−0.81	−4.57	<0.001
F_14-d flexural_	Constant	7.73	0.688	/	8.09	<0.001
	A/P	−0.96	0.29	−0.69	−4.27	0.001
	W/P	−3.44	0.733	−0.86	−1.77	0.024
F_28-d compressive_	Constant	25.04	0.471	/	16.38	<0.001
	A/P	−2.4	0.198	−0.34	−5.236	<0.001
	W/P	−16.3	0.502	−0.91	−6.491	<0.001
F_28-d flexural_	Constant	7.15	0.921	/	7.048	<0.001
	A/P	−0.88	0.388	−0.638	−1.57	0.035
	W/P	−2.54	0.981	−0.562	−3.245	0.005

## Data Availability

Some or all data and code that support the findings of this study are available from the corresponding author upon reasonable request.
